# New imaging agents targeting chemokine receptor CXCR4 for PET/SPECT and MRI

**DOI:** 10.1186/2197-7364-1-S1-A81

**Published:** 2014-07-29

**Authors:** Sophie Poty, Pauline Désogère, Kati Nicholson, Shubhanchi Nigam, Christine Goze, Frédéric Boschetti, Steve Archibald, Helmut Maëcke, Franck Denat

**Affiliations:** ICMUB (UMR CNRS 6302), 9 Av. Alain Savary, BP 47870, 21000 Dijon cedex, France; Department of Chemistry, The University of Hull, Kingston upon Hull, HU6 7RX UK; Chematech, 9 Av. Alain Savary, BP 47870, 21000 Dijon cedex, France; Universitätsklinikum Freiburg, IMSRobert-Koch-Str. 1, Freiburg, D-79106 Germany

CXCR4 is a chemokine receptor overexpressed in numerous cancer types that plays a key role in invasion and metastases. For this reason, CXCR4 appears as an interesting biomarker for the field of diagnostic oncology and in the past few years it has gained interest for application in molecular imaging. Both peptide-based and small molecules have been developed as CXCR4 inhibitors. Among all existing organic molecules, cyclam derivatives AMD3100 and AMD3465 were described as strong CXCR4 inhibitors. To image CXCR4 expression in tumor models, macromolecular agents such as ^111^In- and ^18^F-labeled peptides and ^125^ I-labeled monoclonal antibodies have been investigated using either SPECT or PET. Recently, AMD3100 and AMD3465 were directly radiolabeled with ^99m^Tc and ^64^Cu, but these complexes have a low in vivo stability leading to the decomplexation of the metal. In this context, we decided to use the high affinity of these molecules for CXCR4 and to attach to these systems an optimized chelating agent in order to image CXCR4 by SPECT/PET imaging or MRI using different metals such as ^111^In, ^64^Cu, ^68^Ga or Gd.

AMD3100 and AMD3465 were modified in order to introduce an adapted chelator. Our new analogues were then metalated with Gd, cold Ga, In, and Cu, but also ^68^Ga and ^64^Cu. Biological experiments on Jurkat and MCF-7 cell lines were performed to determine the affinity of our new imaging agents toward the CXCR4 receptor.

We will present the synthetic pathway toward our new AMD analogues together with flow cytometry and radiolabelling experiments. Two compounds show promising results and will be used for *in-vivo* experiments.

We report here new imaging agents targeting the chemokine receptor CXCR4 that could be use either for PET/SPECT and MRI. Our concept also offers the possibility of attaching a fluorescent tag for optical imaging or bimodal systems.

